# Co-Measure: developing an assessment for student collaboration in STEAM activities

**DOI:** 10.1186/s40594-017-0094-z

**Published:** 2017-11-15

**Authors:** Danielle Herro, Cassie Quigley, Jessica Andrews, Girlie Delacruz

**Affiliations:** 1 Digital Media and Learning, College of Education, Clemson University, Clemson, SC 29634 USA; 2 Science Education, Clemson University, Clemson, USA; 3 Educational Testing Service, Princeton, NJ, USA; 4 LRNG, Chicago, IL, USA

**Keywords:** STEAM instruction, Collaborative problem solving, K-12 education and STEAM, STEAM assessment

## Abstract

**Background:**

The shortage of skilled workers choosing STEM (Science, Technology, Engineering, and Math) careers in the USA and worldwide has fueled a movement towards STEAM, in which the “A” addresses the arts and humanities. STEAM education has been proposed as a way to offer relevant problems to solve while drawing on creative and collaborative skills to increase interest and engagement in these fields. Despite the interest in increasing STEAM globally, research on the efficacy of instructional approaches, including ways to assess collaborative problem solving (CPS), is lacking.

**Results:**

This paper reports the development of a rubric, named Co-Measure, for researchers and educators to use to assess student collaboration, at the individual level, when students are working in K-12 STEAM activities. Our project team provides the rationale, process, validation, initial iterations to the rubric, and next steps to inform STEM researchers and move STEAM instruction and learning forward. A final rubric is provided and made freely available to researchers and educators.

**Conclusions:**

As STEAM education gains popularity in K-12 schools, assessment of student collaboration is needed to identify the dimensions of the skill in order to provide appropriate problem solving opportunities within instruction. The assessment is also needed to adjust instruction when students are not offered opportunities to work collaboratively during STEAM activities. Utilizing existing generalized frameworks of CPS provided the initial guide to direct research specific to CPS in STEAM activities. Using an iterative process to identify and evaluate attributes of student behavior associated with CPS in STEAM classrooms by a project team comprised of learning scientists, educational researchers and psychometricians allowed for rigorous research while drawing on appropriate expertise. Co-Measure has the potential to be modified and broadly extended to assess CPS in elementary and post-secondary classrooms using STEAM instructional practices.

**Electronic supplementary material:**

The online version of this article (10.1186/s40594-017-0094-z) contains supplementary material, which is available to authorized users.

## Findings

### Introduction

In the 1990’s, the National Science Foundation identified subject areas crucial to improving economic development within the USA, heightening the importance of Science, Technology, Engineering, and Math (STEM) education (Portz 2015). The last two decades have seen numerous efforts towards developing specialized STEM programs within hundreds of middle and high schools across the country, as well as demands to increase funding for specialty STEM high schools—yet the USA has seen relatively small increases in young men and women choosing STEM fields in higher education and the workforce (Atkinson et al. 2007; Chen 2013). A current movement is underway to attract today’s young learners to STEM by emphasizing the arts in K-12 education (Bequette and Bequette 2012). STEAM, where the “A” represents both the arts and humanities, has been proposed as an engaging and more realistic learning experience than STEM because the arts and humanities purportedly allow for better integration of the disciplines while encouraging creativity and problem solving (Gettings 2016; Miller and Knezek 2013). School-wide programs to offer STEAM education are increasing each year and have been adopted in a handful of schools around the USA including California, Virginia, North Carolina, South Carolina, Georgia, Massachusetts, Texas, and throughout Australia, Korea, and some European countries (Quigley and Herro 2016; Delaney 2014). The New Media Consortium’s Horizon Report describes STEAM as a rising trend in K-12 education, predicting it will likely be adopted in many K-12 schools in less than 3 years (Johnson et al. 2015).

STEAM has also been conceptualized as a transdisciplinary teaching and learning approach where you start with the “issue or problem and, through the process of problem solving, bring to bear the knowledge of those disciplines that contributes to a solution or resolution” (Meeth 1978, p. 10). Transdisciplinary approaches differ from multidisciplinary approaches, commonly used in STEM teaching, in that multidisciplinary approaches typically begin with a discipline or multiple disciplines and then instructors create challenges for students to solve. Wickson et al. (2006) further explain transdisciplinary teaching as problem oriented, while multidisciplinary teaching is often organized thematically (e.g., biophysics or mathematical engineering). To that end, STEAM instruction has been conceptualized as foregrounding the problem to be solved by using (1) project-based learning; (2) technology to some extent for creativity and design; (3) inquiry approaches, allowing multiple paths to solve a problem; (4) science, technology, engineering, arts/humanities, and math as required by the problem; and (5) collaborative problem solving (Herro and Quigley 2016). Instructors use transdisciplinary approaches in a holistic manner and present overarching idea or problems relevant to students’ locale and lives, in which students form collaborative groups exploring and designing solutions to an open-ended problem.

Jolly (2014) addressed the tension between STEM and STEAM advocates while attempting to disentangle the difference between the two concepts. She suggested that both have merit but that STEM focuses more heavily on math and science and encourages teamwork more than collaboration, while STEAM might consider the “arts” broadly to include design, computer graphics, performing arts, creative thinking, or even playful problem solving when exploring and designing solutions. STEAM may also include elements of design education wherein creative processes are used to “study aesthetics and utility of items in our daily lives” (Vande Zande 2010, p. 249). STEAM also differs from project-based STEM teaching, often referred to as integrative STEM, both may encourage collaboration; however, in many classrooms integrative, STEM is still primarily focused on the technological/engineering design aspects of science and math (Ernst and Clark 2007). STEM projects often entail robotics challenges, coding or computer science activities, or interdisciplinary, practical problems such as investigating the science and math behind harnessing wind energy. This differs from the trandisciplinary nature of STEAM where the problem does not necessarily begin with an engineering, math, or science problem, but instead with social issues emulating concerns in the local community (Guyotte et al. 2014). That said, STEAM may also include creative, imagined problems embedded in problem scenarios that may not appear plausible but serve to assist students in conceptualizing and solving the larger, authentic problem. STEAM curricula *are different* than other comprehensive curricula in that trandisciplinary approaches are seldom used in classrooms. This is because they require expertise across content areas, involve authentic problem creation based on students’ locales and interests, and are difficult to implement (Quigley and Herro 2016).

National and global interest in STEAM education is growing, and predictive reports suggest the trend will likely continue; however, there is surprisingly little research on the efficacy of instructional approaches. Furthermore, there is a lack of research in determining and measuring essential 21st century skills (e.g., creativity, innovation, communication, collaboration) important for success in STEAM activities (Platz 2007). Our research addresses ways to assess one important skill in STEAM, collaboration. Similar to STEM, collaboration is typically embedded in the problem solving process and critical to honing negotiation skills to arrive at potential solutions (Land 2013).

While interest in measuring collaboration has increased with access to collaborative Internet technologies, past efforts to develop models to assess collaboration have typically occurred in organizations addressing healthcare, business, or higher education (Thomson et al. 2009). This paper aims to articulate the development of a rubric for researchers and teachers to use to assess student collaboration, at the individual level, when working in K-12 STEAM activities.

### Defining collaboration for problem solving

Collaboration has been broadly defined as a “coordinated, synchronous activity that is the result of a continued attempt to construct and maintain a shared conception of a problem” (Roschelle and Teasley 1995, p. 70). There is a growing interest in the assessment of skills associated with collaboration, particularly in problem solving situations, a critical component of STEAM teaching, given the importance of collaboration in a variety of contexts including the school, workplace, and military. Advantages of collaboration for problem solving have been attributed to the fact that collaboration affords more effective division of labor, the incorporation of solutions from group members with differing perspectives, knowledge, and experience, and enhanced solution quality by the ideas of other group members (Graesser et al. 2017).

Assessment research defines the construct of collaborative problem solving (CPS) as well as examines particular observable behaviors indicative of competencies associated with the construct. For example, the Organisation for Economic Co-Operation and Development developed a CPS framework for implementation for the Programme for International Student Assessment (PISA). The PISA 2015 CPS framework identified three core CPS competencies: establishing and maintaining shared understanding, taking appropriate action to solve the problem, and establishing and maintaining team organization. The framework further crossed the three CPS competencies with four problem solving processes (i.e., exploring and understanding, representing and formulating, planning and executing, and monitoring and reflecting) to create a matrix of skills. The PISA CPS matrix includes skills such as discovering perspectives and abilities of team members, identifying and describing tasks to be completed, and monitoring, providing feedback, and adapting the team organization and roles (Organisation for Economic Co-operation and Development 2013).

Additional frameworks conceptualize collaboration in several ways (e.g., Griffin et al. 2012; O’Neil et al. 2003; Trilling and Fadel 2009). A very recent example developed at Educational Testing Service (ETS), in contrast to the PISA CPS framework, does not try to separate cognitive and social dimensions, but instead focuses on the integrated functions of both dimensions in supporting the building of knowledge. Furthermore, rather than focusing on CPS as a construct, ETS seeks to provide a broader assessment tool of CPS focused on eliciting individual and group cognition. The ETS CPS framework includes four CPS skills: sharing resources/ideas, assimilating and accommodating knowledge/perspective taking, regulating problem solving ideas, and maintaining positive communication (Liu et al. 2015).

Furthermore, computer-supported collaborative learning (CSCL) researchers developed alternative conceptualizations of collaboration assessment (Gweon et al. in press; Rummel and Spada 2005; Spada et al. 2005) and methods of analyzing collaborative processes such as ethnographic studies (Koschmann et al. 2003) and automated analyses (Dönmez et al. 2005; Rosé et al. 2008).

For example, Meier et al. (2007) developed a rating scheme that identifies dimensions of collaboration processes in order to determine the quality of CPS in a broad range of computer-supported contexts. The Meier et al. rating scheme included five aspects of collaborative process and corresponding dimensions for each collaborative process. Specifically, they included communication (sustaining mutual understanding, dialog management), joint information processing (information pooling, reaching consensus), coordination (task division, time management, technical coordination), interpersonal relationship (reciprocal interaction), and motivation (individual task orientation).

The CPS frameworks developed explicate a number of skills relevant for collaborative problem solving, pulling from literature across many research areas, such as computer-supported collaborative learning, individual problem solving, and team discourse analysis. Many frameworks, however, present the CPS skills in a general manner removing all contexts (i.e., what students would experience during instructional units in classrooms) from the operationalization of the construct. This sort of operationalization is appropriate for the assessment purposes of entities such as PISA and ETS in creating a means by which to assess individuals’ collaborative skills in large-scale computerized contexts and the purposes of much CSCL research in developing generic assessment methods. However, in smaller scale classroom contexts, there are often educational approaches that incorporate collaboration and are specific to requisite content. As a result, there is a need for CPS frameworks that identify CPS skills specific to particular content areas and domains. We address this need by developing an assessment tool, Co-Measure, which identifies CPS skills specific to STEAM.

### Rationale and purpose of Co-Measure

Collaboration can improve learning outcomes through a number of mechanisms, including opportunities for verbalizing and elaborating one’s own ideas and resolving potential discrepancies with peers to facilitate critical thinking (Andrews and Rapp 2015). These benefits are particularly useful in STEM fields, which often involve individuals with diverse perspectives working together to solve complex problems. A number of studies have demonstrated gains in student understanding of STEM content when given the opportunity to engage in discussion with peers (Barron 2000; Smith et al. 2009; Whicker et al. 1997).

Co-Measure is a rubric focused on evaluating student CPS when participating in STEAM activities, tasks, or units of study. The development of Co-Measure acknowledges collaborative skills are crucial for all learners in the digital age and addresses the increasing need to validate efforts towards enhancing young people’s skills when engaging in collaboration. While there are many initiatives aimed at designing and implementing curricula to promote collaboration, there has not been equivalent effort devoted to assessment tools that utilize advances in assessment practices, particularly those that are shown to facilitate the assessment of complex skills (Mislevy 2013). The development of Co-Measure was also, in part, to address a need identified by classroom teachers engaged in a prior multi-year study exploring STEAM instructional practices (Quigley and Herro 2016). One result of the study found teachers unable to distinguish, and consequently asking for a distinction, between group work (where students are placed in groups to work together) and productive collaboration. Thus, Co-Measure has two goals aimed at assisting researchers and teachers: to identify collaborative skills specific to STEAM activities and to assess student collaboration during STEAM learning at the individual level.

### The process

Our project team is comprised of learning scientists, educational researchers, and psychometricians from universities, ETS and a National Center for Research. The educational researchers were engaged in the previously mentioned STEAM research and suggested addressing the need to assist researchers and teachers in determining how to identify and assess collaboration in STEAM activities. Throughout the process, the project team consulted with mentors from ETS and learning scientists serving as advisors to the project. Mentors provided feedback on all aspects of the process and rubric.

The team’s process for creating the rubric can be summarized as (1) reviewing literature and existing CPS frameworks, (2) collecting video data to examine instances of students working on STEAM classroom activities, (3) discussing potential indicators of STEAM CPS based on STEAM instructional practices and desired student behaviors, (4) determining pre-existing and emerging themes based on the indicators and further analysis of video data to define attributes, (5) aligning behaviors from the video data to the indicators until researcher consensus was reached, (6) testing the rubric in STEAM classrooms, (7) refining the rubric with the project team, and (8) validating and iterating the rubric. It is important to note that the process was not always sequential; at times, portions of the work progressed simultaneously or steps were repeated as the rubric was iterated. Below, we explain the significant components of the process in greater detail.

#### Reviewing existing literature and CPS frameworks

The project team reviewed existing literature relevant to collaboration to explore various components of collaboration that would be important for assessment purposes. The project team explored areas of research such as computer-supported collaborative learning, social psychology, psycholinguistics, team assessment, and cognitive science. These literatures allowed for the exploration of work related to things such as individual problem solving, discourse analysis, and organizational teams. Existing CPS frameworks were also identified and evaluated to determine skills and processes related to collaboration that were relevant for STEAM activities in educational settings. These frameworks included, among others, CSCL collaboration schemes (e.g., Meier et al. 2007), the PISA CPS Framework (Organisation for Economic Co-operation and Development 2013), the ETS CPS Framework (Liu et al. 2015), the Assessment and Teaching of 21st Century Skills CPS Framework (Hesse et al. 2015), the CRESST teamwork process model (O’Neil et al. 2003), and other models of teamwork (e.g., Morgan et al. 1993; Stevens and Campion 1994; Zhuang et al. 2008).

#### Video data collection

Two members of the project team collected video data from seven different middle school classrooms in the southeastern USA in which teachers were implementing STEAM activities or units of study. Prior to this STEAM instruction, teachers had participated in 50 h of professional development, which included instruction on problem-based learning, transdisciplinary teaching practices, integrating technology, and creating STEAM units. As a follow-up to the professional development, teachers were observed and given constructive feedback regarding their efforts to implement STEAM activities or units (see Appendix for a sample STEAM unit). During these observations, video data from each site were collected. Researchers focused closely on students working in groups of typically three to five students and a wireless microphone was placed in the center of each group to capture verbal exchanges.

#### Identifying indicators of STEAM collaboration practices

During the literature review, a number of behaviors associated with collaboration were identified and used as a priori themes. For example, some of the noted CPS behaviors included students making choices, providing feedback to one another, moving from self-reliance to group reliance, and negotiating roles. Additionally, the prior 50-h professional development (Quigley and Herro 2016) resulted in identifying effective instructional practices, which were in turn used as themes that might elicit particular student behaviors. The instructional practices (themes) included authentic tasks, inquiry rich, student choice, and student-directed learning. That is to say, teachers enacting STEAM units are encouraged to offer students problems to solve that are closely related to real world problems and opportunities to explore a variety of solutions through self-directed learning and inquiry. Thus, to analyze the video data and identify potential indicators of collaboration, the research team first began by using the pre-determined themes from the literature review and instructional practices and then looked for instances in which students displayed those behaviors to assist in more fully describing or modifying them. Transana (https://www.transana.com/), a transcription and analysis software for audio and video data, was used to identify and code instances of behaviors relating to the themes; this was repeated until the project team achieved consensual validation (Creswell 2007). Finally, from these themes, indicators, or “attributes,” of the behaviors were defined and grouped into “dimensions” of collaboration.

For example, in the case of student-directed learning (eventually part of a dimension defined as “Peer interactions”), during video analysis, researchers observed students dividing the tasks, assisting and redirecting one another to complete work, checking for understanding, and at times providing peer feedback. In instances where students were given an authentic task, we sometimes observed students connecting relevant, prior knowledge to assist others in approaching the problem or using collaborative (technology or non-digital) tools that mimicked what real-world scientists or other professions might use. Thus, a dual approach of first relying on prior literature and instructional practices, and then observing student practices within the video data, allowed us to determine and refine specific indicators. Again, these indicators became the attributes and dimensions (see next section) that were refined for each draft of the rubric.

Ultimately, the project team determined five dimensions based on the literature review, instructional practices, the video data, and desirable collaborative behaviors. The original dimensions consisted of (1) Student-directed Learning, (2) Positive Communication, (3) Inquiry Rich/Multiple Paths, (4) Authentic Approach and Tasks, and (5) Transdisciplinary Thinking. Within each dimension, attributes delineate desired behaviors for researchers and/or teachers to rate individual students as *needs work*, *acceptable*, or *proficient* on each attribute. The dimensions, when compared to other CPS frameworks, are specific to STEAM (e.g., transdisciplinarity is not common in typical comprehensive curricula, nor is the combination of an instructional environment offering all five dimensions). While it might be argued that many attributes are germane to integrative STEM activities, the keen focus during STEAM instruction on students collaboratively exploring inquiry rich/multiple paths in order to encourage them to pose new questions, along with the emphasis on transdisicplinary thinking as they present a variety of solutions to particular problems differentiate the two concepts.

#### Iterating the rubric based on classroom testing

After the first draft of Co-Measure was created, it was tested in three classrooms for usability, which resulted in iterating the rubric. For instance, during the testing, the project team noted the attribute titled “chooses methods or materials” under the dimension, Inquiry Rich/Multiple Paths, would be more accurately represented in the dimension, Authentic Tasks/Approach. Furthermore, this attribute was changed to “negotiates method or materials relevant to solving the problem.” The rationale for this move was the project team noticed that it was when the students were approaching the problem that they began negotiating methods or materials. Additionally, the term “negotiating” was more relevant to the collaborative process, thus motivating the change from “chooses” to “negotiates.” In order to negotiate, students would need to collaborate with someone else, whereas choosing could be a solitary action.

Similarly, on the original rubric, there was a dimension titled “Student-directed Learning,” which included the following attributes: refers to guidelines of rubric to direct work, divides and completes tasks, negotiates roles within group, checks for understanding regarding process and/or content, and provides peer feedback, assistance, and/or redirection. Discussions with collaboration researchers at ETS suggested these attributes could be better characterized more generally as “Peer Interactions,” leading to the renaming of this dimension. An additional issue in implementing the rubric was difficulty in distinguishing the behaviors indicative of the “divides and completes tasks” and “negotiate roles within the group” attributes. To resolve this issue, we modified the descriptors under each proficiency level of these attributes to more clearly distinguish the behaviors corresponding to each attribute.

The attribute, “listens and takes turns,” under the dimension, “Positive Communication,” was originally, “listens and takes turns without interrupting.” However, during classroom testing, the project team noticed that interruptions were sometimes appropriate and did not disrupt, but often enhanced, the collaborative problem-solving process. Therefore, the team modified the acceptable and proficient proficiency levels under the attribute to include “apologizes for inappropriate interruptions.”

Additionally, it was noted that the order of the dimensions would need to be reconsidered. While the collaborative problem-solving process is not linear, the project team realized that it made sense to begin with more general collaborative behaviors and then address increasingly complex behaviors specific to STEAM. The modified order included (1) Peer Interactions, (2) Positive Communication, (3) Inquiry Rich/Multiple Paths, (4) Authentic Approaches/Tasks, and (5) Transdisciplinary Thinking (Additional file 1). However, the project team noted that the user would likely not be able to view these dimensions in sequential order and therefore would need to be able to easily move through the rubric from one dimension to another.

Last, we determined that the current style of the rubric would need to be edited to include a place for users to make notes in order to provide additional feedback to the student, as necessary.

#### Validating co-measure

To validate the rubric, our team developed empirical studies to examine the technical quality of the rubric. We took an argument-based approach to establish validity, which views validity as a process of triangulating evidence and logical analysis to evaluate claims and propositions of an interpretive argument within the constraints of the assessment’s intended interpretations and uses (AERA, APA and NCME 2014). The validity studies occurred across two separate phases. The first phase of activities involved expert review of the rubric and rating the importance of attributes covered in order to ensure that the rubric appropriately measures our targeted construct within the context of extant literature, and as a way of establishing construct validity of the rubric. During the second phase of activities, we ensured usage of rubric was consistent across raters to determine inter-rater reliability.

#### Phase one—construct validity

In phase one, our team mobilized a panel of six experts composed of teachers and one researcher (a former high school teacher) who implemented STEAM units of study using similar pedagogical techniques; three of them had completed the abovementioned 50 h of STEAM PD, two worked in a STEAM school, and all regularly implemented interdisciplinary, authentic, technology-rich, project-based STEAM units. The project team asked the panelists to examine the rubric in detail and provide feedback with regard to coverage and accurate descriptions of collaboration in STEAM. We administered a short survey asking panelists to provide feedback on the five dimensions and supporting attributes with questions to determine if the rubric captured critical attributes (i.e., check for omissions in attributes or if any attributes were irrelevant) and then rate the extent to which they agreed each attribute captured what they might expect to see in each dimension.

To assess construct validity (Wainer and Braun 1988), we added an additional column to the end of each row within the rubric and then asked panelists to individually rate each attribute on a scale of 0–3 in terms of importance with respect to the collaboration construct. The average importance of each attribute was then calculated and the mean was reported. During our analysis, our team began by reviewing items with a mean of 2.0 or less to determine whether the panelists deemed the attribute as a significant aspect of collaboration. Table 1 shows the mean scores of participants’ ratings for each attribute in terms of importance.

**Table 1:** Mean scores of attributes based on perceived importance

Attribute	Rank
Dimension: Peer Interaction	
Monitors tasks/project with peers	3.00 (.00)
Negotiates roles within group	2.33 (.82)
Divides and works toward task completion	1.83 (1.33)
Checks for understanding regarding process and/or content	1.33 (1.03)
Provides peer feedback, assistance, and/or redirection	2.33 (1.21)
Dimension: Positive Communication	
Respects others’ ideas	3.00 (.00)
Uses socially appropriate language and behavior	2.33 (1.03)
Listens and takes turns	1.83 (1.47)
Dimension: Inquiry Rich/Multiple Paths	
Develops appropriate questions towards solving the problem	2.33 (1.21)
Verifies information and sources to support inquiry	2.83 (.41)
Dimension: Authentic Approach and Tasks	
Shares connections to relevant knowledge	2.17 (1.17)
Negotiates method or materials relevant to solving the problem posed	2.00 (1.26)
Uses tools collaboratively to approach task	2.17 (1.17)
Dimension: Transdisciplinary Thinking	
Discusses approaching task, activity, or problem using multiple disciplines	2.00 (1.10)
Co-creates products by incorporating multiple disciplines	2.67 (.52)

Standard deviations appear in parentheses beside means

To confirm if panelists believed that each attribute was a strong indicator of CPS, they were given a second question asking them to indicate the extent to which they agreed that each attribute captured what they might expect to see in each collaboration dimension. They were asked to rate their agreement using a 5-point Likert scale (1 indicating “strongly disagree” and 5 indicating “strongly agree”). All panelists rated 11 of the 15 attributes with agreement that the attribute aligned with the dimension, scoring each a 4 or 5 within the 5-point rating scale. Table 2 shows the panelists’ ratings for how well they believed the attributes aligned with each dimension.

**Table 2:** Rater agreement of attribute alignment with dimension

Attribute	Responses		
	High	Mid	Low
Dimension: Peer Interaction			
Monitors task/project with peers	6	0	0
Negotiates roles within group	6	0	0
Divides and works toward task completion	4	0	2
Checks for understanding regarding process and/or content	3	2	1
Provides peer feedback, assistance, and/or redirection	5	0	1
Dimension: Positive Communication			
Respects others’ ideas	6	0	0
Uses socially appropriate language and behavior	4	2	0
Listens and takes turns	4	1	1
Dimension: Inquiry Rich/Multiple Paths			
Develops appropriate questions towards solving the problem	6	0	0
Verifies information and sources to support inquiry	6	0	0
Dimension: Authentic Approach and Tasks			
Shares connections to relevant knowledge	5	1	0
Negotiates method or materials relevant to solving the problem posed	6	0	0
Uses tools collaboratively to approach task	5	0	1
Dimension: Transdisciplinary Thinking			
Discusses approaching task, activity or problem using multiple disciplines	5	0	1
Co-creates products by incorporating multiple disciplines	6	0	0

High denotes score of 4 or 5 on 1–5 rating scale, medium denotes 3, low denotes 1 or 2

Finally, we conducted interviews with the panelists to establish how they interpreted each attribute and why they selected particular responses. In general, the surveys, ratings, and interviews resulted in eliminating attributes considered redundant, less significant, or irrelevant assisting in validating the constructs and revising the rubric. Table 3 typifies panelists’ comments.

**Table 3:** Example comments from panelists by dimension

Dimension	Example comments
Peer Interactions	C1: There seems to be too many of these. The variations are subtle even from a teacher’s viewpoint. “Negotiates roles within the group” and “Divides work towards task completion” seem very similar. C2:“Monitors tasks/projects with peers” and “Checks for understanding regarding process and/or content” also seem very similar. To clarify it might be beneficial to lump these together.
Positive Communication	C1: Within respecting others ideas you may want to include acknowledging whether ideas were offered at all. C2: This might be the dimension to place something about compromising or taking turns.
Inquiry Rich/Multiple Paths	C1: An attribute might be added that relates to students conducting research including communication with experts in the field. C2: Does this include how students interact when one person decides that there needs to be a change of path?
Authentic Approach and Tasks	C1: I would use some combination of “negotiates methods or materials” and “uses tools collaboratively” as they are closely related. C2: I think the attribute could use an additional research-related dimension since the rubric is assessing the authenticity of the task.
Transdisciplinary Thinking	C1: Some of this could be covered under “positive communication.” C2: Two attributes about multiple disciplines are not necessary.

C1 and C2 indicate separate comments drawn from all 6 participants

Thus, we revised the rubric based on the data collected and analyzed during this first phase. For instance, the dimension of “Peer Interactions” was shortened to include just three attributes, and the dimension of “Transdisciplinary Thinking” was removed entirely as the panelists overwhelmingly believed its two attributes belonged within the dimension of “Authentic Approach and Tasks,” which was relabeled as “Transdisciplinary Approach.” Furthermore, the attribute “negotiates method or material relevant to solving the problem posed” was combined with another attribute and changed to “negotiates roles and divides work to complete tasks,” and the attribute “checks for understanding regarding process and/or content” was added to “monitors tasks/projects with peers” as participants rated them less important than other attributes, and they suggested combining them in survey and interview data.

Minor wording changes were made to clarify attributes such as adding, “comprises” to “respects others’ ideas” to currently read “respects others’ ideas and comprises” based on surveys and interviews.

#### Phase two—reliability of ratings

During phase two, we convened the same team of six experts to serve as raters using the revised version of Co-Measure. The process began with the panelists again receiving training on how to use Co-Measure, including written descriptions to define potentially ambiguous terms such as “occasionally,” “usually,” “multiple paths,” and “co-creates.” During this phase of the study, our aim was to establish consistency *within* dimensions and *across* dimensions.

##### Panelist ratings within dimensions

First, panelists watched 19 video clips, from seven classrooms, highlighting students working together within a variety of STEAM units. The videos were shown as anchor exemplars, depicting each attribute, and purposely included portrayals of each dimension with a variety of attributes and potential ratings (*needs work*, *acceptable*, or *proficient*). Raters watched the video clips and then independently scored each clip according to attributes within one dimension. This was followed by a discussion to determine if they could reach consensus on ratings within each dimension and assign a final score. A member of the project team tracked the scores and noted the discussion.

During this activity, panelists were able to reach consensus when using the rubric to score every video. They also agreed that two of the attributes (i.e., “develops appropriate questions and methods towards solving the problem” and “discusses and approaches problem solving incorporating multiple disciplines”) were not readily apparent in the video data, despite our efforts to offer anchor exemplars. In this case, half of the panelists scored them and the other half did not, and after a discussion, their consensus was the attributes were not obvious in the clips. In these two instances in which panelists did not provide a score for the attribute, the discussion focused on whether the attribute *could* be easily observed in a classroom. Panelists felt the attributes could be observable in their classrooms and provided examples such as students brainstorming their plan and methods when beginning a new project, building something and then adjusting the structure, or creating an object during a forces and motion unit that has historical significance and then talking about how they might use mathematical equations or angles to solve the problem. Thus, the panelists did agree that the attributes could be easily observed and scored. Finally, we calculated the inter-rater reliability for the attribute ratings to determine consistency among raters and found Fleiss kappa = .65, indicating substantial agreement (Landis and Koch 1977).

##### Panelist ratings across dimensions

Next, the panelists individually rated two longer video clips to determine consistency across dimensions. They were provided the entire rubric and were asked to decide which dimension the clip belonged to. Within the dimensions of Peer Interaction, Positive Communication, and Inquiry Rich/Multiple Paths, all of the panelists were able to identify and score each attribute within the dimensions for both video clips. Two of the attributes within Transdisciplinary Approach were also scored by all six panelists, and two other attributes, “discusses and approaches problem solving incorporating multiple disciplines” and “negotiates relevant method or materials to solving the problem posed”, were not apparent to four of the six and six of the six panelists, respectively. That is, all panelists made the same scoring choices for every dimension except for two attributes within Transdisciplinary Approach. Data from follow-up discussions and interviews showed that panelists believed the videos lacked an in-depth view of students “having discussions” or “negotiations” related to those two attributes, so they did not score them.

Additionally, survey and interview questions asked panelists to rate the rubric’s ease of use and suggest recommendations for improvement. Panelists were also asked to rate their ability to understand terms, use and score all of the dimensions, to reach consensus, and whether the second iteration of the rubric appropriately assesses CPS in STEAM activities. In general, they responded that they had little difficulty learning to use the rubric, the instructions to clarify terms were helpful and important, reaching consensus was not difficult, and they experienced very little difficulty in using the rubric to individually determine a score. Using a Likert scale (1 = very poorly, 5 = very well) to indicate to what extent they believed the rubric appropriately assesses CPS in STEAM activities, five panelists rated it as 5, with just one panelist rating it as 3. In follow-up interviews, that panelist expressed hesitancy over the length of the rubric. He admitted that he found it easy to use while scoring students in the videos, however he was not sure how accurately he could assess an entire classroom of students and therefore was unsure if it would appropriately assess CPS in STEAM activities within his classroom.

Importantly, most panelists noted that having the context of their own classroom with more time, instead of using short video clips, would likely make the rubric easy to use in terms of seeing and scoring the various attributes. The panelists made two recommendations, which emerged from survey and interview data during both phases. First, they recommended the rubric would need to be shortened to be useful for teachers (not necessarily for researchers or during peer assessment) who were teaching while assessing students, considering the pace of classroom teaching. Second, panelists recommended a companion rubric in student-friendly language that would be helpful for students’ self-assessment. The process outlined above resulted in the current version of Co-Measure (see Additional file 2), which was again shared with the expert panel to verify it reflected their evaluation.

### Discussion

Developing and iterating a rubric to accurately assess student collaboration in STEAM activities is an arduous but important task when considering the nascent nature of CPS frameworks to inform the work (Organisation for Economic Co-operation and Development 2013) and number of K-12 schools adopting STEAM initiatives (Quigley and Herro 2016). The development of Co-Measure addresses the need to identify and assess collaborative problem-solving skills specific to STEAM activities. Drawing on literature from various domains (e.g., collaborative learning, discourse analysis, organizational psychology) and existing CPS frameworks, as well as examining instances of students working in STEAM activities aligned with a priori themes, allowed our team to develop an initial rubric. Small-scale usability testing in STEAM classrooms and meetings with mentors yielded a second draft of Co-Measure that was validated through empirical studies, guiding the rubric’s refinement and improvement. One area, in particular, proved difficult for panelists to rate from the video clips. This was determining if students were discussing and approaching their problem solving incorporating multiple disciplines. In follow-up interviews, panelists talked about the importance of keeping the attribute on the rubric and the need to consciously and consistently set up STEAM problem-solving scenarios that were transdisciplinary in nature, foregrounding the problem but drawing on multiple disciplines (Wickson et al. 2006) and offering overt roles for students to discuss the problem as someone in real world might do. For example, a STEAM problem focused on a spacecraft safely re-entering the earth’s atmosphere might have students take on the role of manager, mission control expert, ground controller, and astronomer, with each bringing a different aspect of problem solving to the collaboration, creating a need to have a discussion focused on multiple disciplines.

Feedback from the panelists using the rubric also provided important information about ease of practical use, whether any gaps with respect to attributes were not accounted for and whether the attributes presented are relevant for STEAM educational contexts. This feedback facilitated further refinement of the rubric, and in turn, the updated rubric will inform our future professional development with teachers, as a portion of STEAM professional development involves formative assessment in STEAM units. Along with assessing students’ content mastery, the formative assessment includes assessing collaborative work, which in prior professional development could only be done anecdotally, which meant that it was done informally based on the teachers’ personal thoughts about what collaboration meant. Further, from the outset of planning instruction, Co-Measure can help teachers identify collaborative skills specific to STEAM activities and plan and practice instructional techniques offering students opportunities to collaborate.

Validating the rubric also demonstrated that the various problems posed to students within STEAM activities, compounded by the contextualized nature of learning and instruction in K-12 classrooms (where students, structure and resources vary greatly), complicate effective use of the rubric. One way to address consistent use of the rubric is ensuring a common understanding of how to use the rubric across contexts. To that end, separate researcher and teacher instructions for use have been developed with setup tips, explanations and definitions for the dimensions and attributes of the rubric, and suggestions for how to use the observation notes provided within each dimension. Setup tips include instructions for video-recording instances of student collaboration, capturing verbal interactions, noting the problem or scenario being worked on, suggesting the length of each observation, and avoiding disruptions as students are working.

The development of Co-Measure and its use was limited to students in 6th–8th grade; a larger study examining a variety of teaching environments and grade levels implemented across diverse populations is necessary to determine the generalizability of our findings. Our next steps include testing Co-Measure in wider variety of elementary and middle school classrooms, aimed at more diverse student populations where teachers have participated in STEAM professional development. Finally, we intend to modify the rubric for use as self and peer assessments. This may entail minor wording changes (e.g., changing *diplomatically* to *tactfully* under the dimension “Positive Communication”). It will also require simplifying the rubric so that it is easily understood and succinct enough for student use.

### Conclusion

As STEAM education gains popularity in K-12 schools, assessment of student collaboration is needed to identify the dimensions of the skill in order to provide appropriate problem solving opportunities within instruction. The assessment is also needed to adjust instruction when students are not offered opportunities to work collaboratively during STEAM instruction. In this case, utilizing existing generalized frameworks of CPS provided the initial guide to direct research specific to CPS in STEAM activities. Using an iterative process to identify and evaluate attributes of student behavior associated with CPS in STEAM classrooms by a project team comprised of learning scientists, educational researchers, and psychometricians allowed for rigorous research while drawing on appropriate expertise. This research answers the call to determine and measure essential 21st Century Skills—skills that include collaboration (Platz 2007). Importantly, it will provide a model for K-12 researchers and teachers for assessing student CPS when engaged in STEAM activities.

### Additional files

Additional file 1: Co-Measure Rubric Before Expert Panel Validation. (PDF 399 kb)

Additional file 2: Co-Measure Rubric After Expert Panel Validation. (PDF 252 kb)

## Appendix

**Table 4:** Abbreviated example of 8th grade science STEAM unit

Project scenario: As more people move to Charleston and businesses continue to grow, traffic is increasing and causing delays, especially along Clements Ferry Road. For example, this means that a person driving 10 miles from their home on Daniel Island to Wando High School might spend 45 minutes in traffic for what should take less than 20 minutes. This comes at a great cost to families who spend less time together, the environment that becomes polluted by increased and slower traffic, and the economy as work time is reduced. As a city planner, you have been given the challenge of designing a solution to the traffic problems in the local area. City planners have to use the scientific and engineering design process in order to find and test solutions, luckily you know these strategies! You have decided it will be helpful to think about the history of South Carolina and how it has changed since colonial times to get a better idea of the land and transportation needed. Also, you will research and read articles on the geographic area, traffic, and the increased houses and businesses expected for Clements Ferry Road. You will need to propose a plan to alleviate traffic issues along with a model to show the city council, and you will also need to be prepared to share the information with the community.
Driving question: How can studying history and traffic patterns in a region help to design a solution to transportation problems?
Elements of STEAM
Science: Students will explore point and non-point pollution sources examining how adding permeable or non-permeable surfaces can affect water flow. Students will model this water flow with permeable and non-permeable surfaces.
Technology: Students will use design software (SketchUp), Chromebooks (Google Apps and Research), and iPads or iPhones to record videos. They will also explore VR technology in the SS class and use Adobe Spark to create and narrate videos describing ‘forgotten’ cities.
Engineering: Students will be using the engineering design process to plan and then design a model of the traffic flow, including examining issues with water flow, etc.
Arts: Students examine emotional and aesthetic impact of changing the streets and how it will affects homes, churches, and the local community. Students will investigate “forgotten” cities in the area and Route 66, and the impact on cities. They will create a mural for the neighborhood affected by this construction. Students will consider the impact on nature and propose ways to keep the area aesthetically pleasing and natural with the trees, river, and animals.
Mathematics: Students will explore the increase in population and its impact on homes. Ratio, scale and proportion will be used to develop their model. They will also develop a budget for this project and use graphing software.
Content Standards
English Language Arts
1. Write arguments that: a. introduce claims, acknowledge and distinguish the claims from alternate or opposing claims, and organize the reasons and evidence logically; b. use relevant information from multiple print and multimedia sources; c. support claims; d. use an organizational structure that provides unity and clarity among claims, counterclaims, reasons, and evidence using valid reasoning and a variety of relevant evidence from accurate, verifiable sources; e. develop the claim and counterclaims providing credible evidence and data for each.
2. Employ a critical stance to demonstrate that relationships and patterns of evidence lead to logical conclusions, while acknowledging alternative views.
3. Develop a range of questions to frame inquiry for new learning and deeper understanding.
Social Studies
1. Summarize the history of English settlement in New England, the mid-Atlantic region, and the South, with an emphasis on South Carolina as an example of a distinctly southern colony.
2. Summarize key economic issues in present-day South Carolina.
3. Explain the tensions between the Upcountry and Lowcountry of South Carolina, including their economic struggles after the Revolutionary War, their disagreement over representation in the General Assembly, the location of the new capital, and the transformation of the state’s economy.
Science Standards
1. Apply scientific principles to design a method for monitoring and minimizing a human impact on the environment.
2. Construct an argument supported by evidence for how increases in human population and per-capita consumption of natural resources impact Earth’s systems
Brief description of students’ activities
Day 1: Introduction of scenario.
Day 2: Students will make a historical timeline describing the population changes that took place in SC.
Day 3: Students will analyze maps, charts, and timelines to further understand changes in SC’s population, economy, and movement. Students will conduct a close read of newspaper articles on the traffic issues that Charleston residents face, specifically in the Clements Ferry corridor. Students conduct research, brainstorm ideas towards their solutions, discuss them and then get ‘scenario’ cards with proposed solutions that they have to discuss in terms of pros/cons and social issues. Solutions they might discuss could include more public transportation (a controversial issue because of the financial cost), working from home 2 days a week, halting/limiting development, mandating carpooling, taxing SUVs etc. Students must equip their proposal with claims, counterclaims, evidence, and rebuttals.
Day 4: Students will use Recap, Kahoot, and Quizlet to check for their understanding of population changes in the area. Begin design process.
Day 5: Students will use the VR glasses to see how the location of different places impacts population and transportation. Students will write a script of questions to use during the interviews. Students will conduct interviews with parents, teachers and other members of the community to find out their opinions of the traffic and how to fix the traffic impacts them and ways to fix it. Students can either record the interviews in video format, podcast format, or written (presentation). The interviews will influence their decision making process of their proposal.
Day 6-7: Students will use their knowledge of SC history to support their design and plan. They will make a presentation to share with the class aligned with their design and proposal in Science and ELA. Students will finalize their videos and their talking points for their presentation. Begin murals.
Day 8: Students will present their designs to the council (a panel of community members and/or teachers)
Day 9-10 – Work on completing murals.
Resources/Equipment needed
Chromebooks - for Sketchup and access to collaboration platforms and Google Drive, iPhone, iPad or some other device that records videos., VR Google Expedition Kits, Newspaper articles from the local area on traffic, etc., Proposal Outline or Template, sketchbooks, paint, art supplies for mural
Experts
City planner, community members, law enforcement officer, arts council member
Technology Integration
Sketchup Make (a free version) to design the plan for the road construction https://www.sketchup.com/, Chromebooks and Google Apps will be used to research, collaborate, and create docs/forms/graphs etc. Virtual Reality Google Expedition Kits
Assessment
Formative
Recap can be used to have students reflect on the varying steps of the unit. Kahoot will be used to check for understanding of the Scientific Method and/or Engineering Design Process. Review of interview questions. Check-ins and quizzes for embedded math skills.
Summative
Historical Timeline of the Changes in South Carolina - S.S. Proposal written to the council – ELA; Model on water flow – Science; Visual slideshow and mural critique-Art.
