# Supplementary material for: Co-Measure: developing an assessment for student collaboration in STEAM activities
Source: Int J STEM Educ. 2017 Nov 15;4(1):26. doi: 10.1186/s40594-017-0094-z (PMC6310374; doi:10.1186/s40594-017-0094-z)
Supplement: Supplementary file 1 — Co-Measure Rubric Before Expert Panel Validation. (PDF 399 kb) [file 40594_2017_94_MOESM1_ESM.pdf]

Co-Measure

A Rubric to Assess Student Collaboration in STEAM Units

INFORMATION

Complete the fields below:

Name(s)

Date

Classroom Information

DIRECTIONS

The following rubrics are designed to be edited digitally or marked and annotated after printing

Dotted lines enclose editable areas in which to type comments

The ✓ icon denotes a clickable area to be selected or marked

SAMPLE STEAM SCENARIO

Write a brief description of your STEAM scenario in the space below:

1 PEER INTERACTIONS

Description

Within STEAM learning, students are expected to refer to the guidelines of the rubric with their peers to identify group goals and monitor progress towards completing the tasks. Group members then discuss how to divide tasks relying on one other's expertise to equitably complete the work. Students rely on their group members to check for accuracy in the process (e.g. Does the way we are approaching the task make sense?) and the content (e.g. Is the content accurate?). Students provide one another with feedback to help them gauge how they are doing or redirect tasks.

ATTRIBUTE	NEEDS WORK	ACCEPTABLE	PROFICIENT
Monitors tasks/project with peers	Student does not rely on peers to discuss criteria, identify goals and monitor progress towards goals ✓	Student occasionally relies on peers to discuss criteria, identify goals and monitor progress towards goals ✓	Student consistently relies on peers to discuss criteria, identify goals and monitor progress towards goals ✓
Negotiates roles within group	Student does not negotiate roles aligned with self/ peer identified expertise, workload is not shared ✓	Student negotiates roles aligned with self/peer identified expertise or attempts to share workload; only one indicator is evident ✓	Student negotiates roles aligned with self/ peer-identified expertise to complete tasks and workload is shared; both indicators are evident ✓
Divides and works toward task completion	Student begins working without discussion of tasks ✓	Student discusses how to divide tasks, but does not always work towards task completion ✓	Student suggests ways to divide tasks and works towards task completion ✓
Checks for understanding regarding process and/or content	Student does not rely on peers to determine accuracy of problem solving process and/or content ✓	Student checks in occasionally with group members to determine accuracy of problem solving process and/or content ✓	Student checks in consistently with group members to determine accuracy of problem solving process and content ✓
Provides peer feedback, assistance and/or redirection	Student does not volunteer or respond to requests to assist in problem solving; provides little or no feedback ✓	Student occasionally volunteers and responds to group member requests; peer feedback is sometimes evident ✓	Student consistently volunteers and responds to group member requests; peer feedback is noted consistently ✓

OBSERVATION NOTES

2 POSITIVE COMMUNICATION

Description

Similar to collaboration in other contexts, positive communication is essential to efficiently work towards solutions. Students participating in STEAM learning are expected to respect one another to foster productive contributions by all members.

ATTRIBUTE	NEEDS WORK	ACCEPTABLE	PROFICIENT
Respects others' ideas	Student rejects others' ideas without an accountable reason	Student occasionally allows others to contribute their ideas; sometimes disagrees diplomatically	Student consistently allows others to contribute their ideas; disagrees diplomatically
Uses socially appropriate language and behavior	Student uses socially inappropriate language and behaviors when interacting with peers	Student occasionally uses socially appropriate language and behaviors when interacting with peers	Student consistently uses socially appropriate language and behaviors when interacting with peers
Listens and takes turns	Student talks over group member, monopolizes conversations or does not talk at all	Student occasionally allows others to finish speaking before he/she speaks; sometimes apologizes for inappropriate interruptions	Student consistently allows others to finish speaking before he/she speaks; apologizes for inappropriate interruptions

OBSERVATION NOTES

3 INQUIRY RICH/ MULTIPLE PATHS

Description

One of the hallmarks of STEAM teaching and learning is that students are given a scenario that has a variety of solutions requiring them to consider various lines of inquiry (or questions) that might arise during task completion. In STEAM collaborative problem solving, we would expect students to work with group members to explore and refine questions, negotiate with group members to choose appropriate materials and methods, and verify information and sources.

ATTRIBUTE	NEEDS WORK	ACCEPTABLE	PROFICIENT
Develops appropriate questions towards solving the problem	Student begins inquiry process without group discussion ✓	Student suggests questions to support inquiry but does not refine questions as he/ she begins problem solving ✓	Student suggests and refines questions to support inquiry towards problem solving ✓
Verifies information and sources to support inquiry	Student does not rely on peers for information or source or verification ✓	Student occasionally checks in with peers to verify information and sources ✓	Student consistently checks in with peers to verify information and sources aligned with inquiry ✓

OBSERVATION NOTES

4 AUTHENTIC APPROACH AND TASKS

Description

STEAM learning assumes students will be presented with problems closely related to local or relevant real-world issues. Throughout group discussions students are expected to regularly share connections to previous experiences, relevant coursework or background content. In STEAM collaborative problem solving, students should be able to discuss and then choose methods or materials that mimic what scientists, researchers, engineers, politicians etc. might do or use. Groups typically use digital and non-digital collaborative tools (e.g. Google Docs, email, whiteboards) to efficiently complete tasks.

ATTRIBUTE	NEEDS WORK	ACCEPTABLE	PROFICIENT
Shares connections to relevant knowledge	Student does not share connections to relevant knowledge including events, places, resources or previous experience ✓	Student occasionally shares connections to relevant knowledge including events, places, resources or previous experience ✓	Student consistently shares connections to relevant knowledge including events, places, resources or previous experience ✓
Negotiates method or materials relevant to solving the problem posed	Student does not negotiate methods or materials, or chooses methods and materials irrelevant to problem posed ✓	Student occasionally negotiates methods or materials, and chooses methods and materials relevant to problem posed ✓	Student consistently negotiates methods or materials, and chooses methods and materials relevant to problem posed ✓
Uses tools collaboratively to approach task	Student uses tools individually ✓	Student occasionally uses tools collaboratively to approach task ✓	Student consistently uses tools collaboratively to approach task ✓

OBSERVATION NOTES

5 TRANSDISCIPLINARY THINKING

Description

A distinguishing characteristic of STEAM learning is that it foregrounds the problem to be solved versus focusing solely on the content and discipline that originated the problem. In other words, collaborative problem solving is used to explore issues and solutions within the problem. Products, such as surveys, posters, videos or other presentations are co-created with attention to multiple disciplines.

ATTRIBUTE	NEEDS WORK	ACCEPTABLE	PROFICIENT
Discusses approaching task, activity or problem using multiple disciplines	Student discusses approaching the task, activity or problem through a single discipline ✓	Student occasionally discusses approaching task, activity or problem through multiple disciplines ✓	Student consistently discusses approaching task, activity or problem through multiple disciplines ✓
Co-creates products by incorporating multiple disciplines	Student does not co-create products demonstrating use of multiple disciplines ✓	Student occasionally co-creates products demonstrating use of multiple disciplines ✓	Student consistently co-creates products that demonstrate use of multiple disciplines ✓

OBSERVATION NOTES
