# Supplementary material for: Co-Measure: developing an assessment for student collaboration in STEAM activities
Source: Int J STEM Educ. 2017 Nov 15;4(1):26. doi: 10.1186/s40594-017-0094-z (PMC6310374; doi:10.1186/s40594-017-0094-z)
Supplement: Supplementary file 2 — Co-Measure Rubric After Expert Panel Validation. (PDF 252 kb) [file 40594_2017_94_MOESM2_ESM.pdf]

Co-Measure

A Rubric to Assess Student Collaboration in STEAM Units

INFORMATION

Complete the fields below:

Name(s)

Date

Classroom Information

SAMPLE STEAM SCENARIO

Write a brief description of your STEAM scenario in the space below:

DIRECTIONS

The following rubrics are designed to be edited digitally or marked and annotated after printing

Dotted lines enclose editable areas in which to type comments

The ✓ icon denotes a clickable area to be selected or marked

1 PEER INTERACTIONS

Description

Within STEAM learning, students are expected to refer to the guidelines of the rubric with their peers to identify group goals and monitor progress towards completing the tasks. Group members then discuss how to divide tasks relying on one other's expertise to equitably complete the work. Students rely on their group members to check for accuracy in the process (e.g. Does the way we are approaching the task make sense?) and the content (e.g. Is the content accurate?). Students provide one another with feedback to help them gauge how they are doing or redirect tasks.

ATTRIBUTE	NEEDS WORK	ACCEPTABLE	PROFICIENT
Monitors tasks and checks for understanding with peers	Student does not rely on peers to discuss criteria, identify goals, monitor progress, and determine accuracy ✓	Student occasionally relies on peers to discuss criteria, identify goals, monitor progress, and determine accuracy ✓	Student consistently relies on peers to discuss criteria, identify goals, monitor progress and determine accuracy; multiple indicators are evident ✓
Negotiates roles, and divides work to complete tasks	Student does not negotiate roles aligned with self/ peer identified expertise, workload is not shared ✓	Student negotiates roles aligned with self/peer identified expertise or attempts to share workload; only one indicator is evident ✓	Student negotiates roles aligned with self/ peer-identified expertise to complete tasks and workload is shared; both indicators are evident ✓
Provides peer feedback, assistance and/or redirection	Student does not volunteer or respond to requests to assist in problem solving; provides little or no feedback ✓	Student occasionally volunteers and responds to group member requests; peer feedback is sometimes evident ✓	Student consistently volunteers and responds to group member requests; peer feedback is noted consistently ✓

NOTES

2 POSITIVE COMMUNICATION

Description

Similar to collaboration in other contexts, positive communication is essential to efficiently work towards solutions. Students participating in STEAM learning are expected to respect one another to foster productive contributions by all members.

ATTRIBUTE	NEEDS WORK	ACCEPTABLE	PROFICIENT
Respects others' ideas and compromises	Student rejects others' ideas without an accountable reason, is unwilling to compromise	Student occasionally allows others to contribute their ideas, and sometimes compromises	Student consistently allows others to contribute their ideas; disagrees diplomatically and compromises
Uses socially appropriate language and behavior	Student uses socially inappropriate language and behaviors when interacting with peers	Student occasionally uses socially appropriate language and behaviors when interacting with peers	Student consistently uses socially appropriate language and behaviors when interacting with peers
Listens and takes turns	Student talks over group member, monopolizes conversations or does not talk at all	Student occasionally allows others to finish speaking before he/she speaks; sometimes apologizes for inappropriate interruptions	Student consistently allows others to finish speaking before he/she speaks; apologizes for inappropriate interruptions

NOTES

3 INQUIRY RICH/ MULTIPLE PATHS

Description

One of the hallmarks of STEAM teaching and learning is that students are given a scenario that has a variety of solutions requiring them to consider various lines of inquiry (or questions) that might arise during task completion. In STEAM collaborative problem solving, we would expect students to work with group members to explore and refine questions, negotiate with group members to choose appropriate materials and methods, and verify information and sources. This often includes a variety of solutions

ATTRIBUTE	NEEDS WORK	ACCEPTABLE	PROFICIENT
Develops appropriate questions and methods towards solving the problem	Student begins inquiry process without group discussion	Student suggests questions and/or methods to support process but does not refine questions and/or methods as he/she begins problem solving	Student suggests and refines questions and/or method to support inquiry towards problem solving
Verifies information and sources to support inquiry	Student does not rely on peers for information or to verify the validity of sources	Student occasionally checks in with peers to verify information and the validity of sources	Student consistently checks in with peers to verify information and the validity of sources

NOTES

4 TRANSDISCIPLINARY APPROACH

Description

A distinguishing characteristic of STEAM learning is that it foregrounds the problem to be solved versus focusing solely on the content and discipline that originated the problem. STEAM learning assumes students will be presented with problems closely related to local or relevant real-world issues. In STEAM collaborative problem solving, students should be able to discuss and then choose methods or materials that mimic what scientists, researchers, engineers, politicians etc. might do or use. Groups typically use digital and non-digital collaborative tools (e.g. Google Docs, email, whiteboards) to efficiently co-create and complete tasks.

ATTRIBUTE	NEEDS WORK	ACCEPTABLE	PROFICIENT
Discusses and approaches problem solving incorporating multiple disciplines	Student does not discuss or approach problem by considering multiple disciplines	Student occasionally discusses and approaches problem demonstrating use of multiple disciplines	Student consistently discusses and approaches problem demonstrating use of multiple disciplines
Shares connections to research or relevant knowledge	Student does not share connections to research or relevant knowledge	Student occasionally shares connections to research or relevant knowledge, which might include events, places, resources or previous experience	Student consistently shares connections to relevant knowledge including events, places, resources or previous experience
Negotiates relevant method or materials to solving the problem posed	Student does not negotiate methods or materials, or chooses methods and materials irrelevant to problem posed	Student occasionally negotiates methods or materials, and chooses relevant methods and materials to problem posed	Student consistently negotiates methods or materials, and chooses relevant methods and materials to problem posed
Uses tools collaboratively to approach task	Student uses tools (digital or non-digital) individually	Student occasionally uses tools (digital or non-digital) collaboratively to approach task	Student consistently uses tools (digital or non-digital) collaboratively to approach task

NOTES
